# What do we want to estimate from observational datasets? Choosing appropriate statistical analysis methods based on the chemical management phase

**DOI:** 10.1002/ieam.4564

**Published:** 2022-01-12

**Authors:** Kazutaka M. Takeshita, Takehiko I. Hayashi, Hiroyuki Yokomizo

**Affiliations:** ^1^ Health and Environmental Risk Division National Institute for Environmental Studies Ibaraki Tsukuba Japan; ^2^ Japan Society for the Promotion of Science Tokyo Japan; ^3^ Social Systems Division National Institute for Environmental Studies Ibaraki Tsukuba Japan

**Keywords:** Akaike information criterion, Biomonitoring, Organic pollution, Statistical causal inference, Trace metal

## Abstract

The goals of observational dataset analysis vary with the management phase of environments threatened by anthropogenic chemicals. For example, identifying severely compromised sites is necessary to determine candidate sites in which to implement measures during early management phases. Among the most effective approaches is developing regression models with high predictive power for dependent variable values using the Akaike information criterion. However, this analytical approach may be theoretically inappropriate to obtain the necessary information in various chemical management phases, such as the intervention effect size of a chemical required in the late chemical management phase to evaluate the necessity of an effluent standard and its specific value. However, choosing appropriate statistical methods based on the data analysis objective in each chemical management phase has rarely been performed. This study provides an overview of the primary data analysis objectives in the early and late chemical management phases. For each objective, several suitable statistical analysis methods for observational datasets are detailed. In addition, the study presents examples of linear regression analysis procedures using an available dataset derived from field surveys conducted in Japanese rivers. *Integr Environ Assess Manag* 2022;18:1414–1422. © 2021 The Authors. *Integrated Environmental Assessment and Management* published by Wiley Periodicals LLC on behalf of Society of Environmental Toxicology & Chemistry (SETAC).

## Key Points

- This article presents an overview of several proper statistical analysis methods for observational datasets in accordance with data analysis objectives within the different management phases of riverine environments threatened by anthropogenic chemicals.
- Examples of linear regression analysis procedures using an available dataset derived from field surveys conducted in Japanese rivers are also presented.

## INTRODUCTION

The protection and restoration of aquatic ecosystems have been a focus of the United Nations' Sustainable Development Goals (United Nations, 2015). Anthropogenic chemicals such as pesticides (Beketov et al., 2013), pharmaceuticals (Richmond et al., 2018), and trace metals (Clements et al., 2000) can impair the integrity of river ecosystems. In such situations, observational datasets (i.e., datasets derived from field surveys) have been used to assess the ecological risks posed by these chemicals (e.g., Iwasaki et al., 2011; Misaki et al., 2019). Observational datasets provide empirical implications for the ecological risks posed by anthropogenic chemicals in the field, which are difficult to obtain using only laboratory toxicity tests (e.g., Cairns, 1988).

For statistical analyses of observational datasets involved in the management of aquatic ecosystems threatened by chemicals, the three major objectives are:

- Identifying sites where biological conditions are severely compromised.
- Screening the chemicals to be investigated in detail and prioritizing.
- Estimating the effect sizes of intervention in the targeted chemical concentration(s).

The first objective can be considered as “where is deterioration occurring?” This information is used to select candidate sites to implement management measures, including those on chemical concentrations. The second objective can be rephrased as “which chemicals should be investigated with high priority?” Many chemicals can contaminate rivers, and with limited funding, screening and prioritization are essential. The first two objectives usually occur in a relatively early management phase for environments threatened by chemical contamination (hereinafter, chemical management). For the last objective, we defined intervention effect size as the amount of change in the outcome variable when the treatment variable is changed by one unit through intervention. An estimation of the intervention effect size is required when evaluating the necessity of introducing management measures (e.g., control of environmental chemical concentrations through setting effluent standards) and their potential intensity. It can be formulated as: “does a reduction in the concentration of the targeted chemical result in the recovery of biological conditions?” and “by how much should the concentration of the targeted chemical(s) be reduced?” Therefore, the last objective typically occurs in a relatively late phase of chemical management. Thus, chemical management has different aims of data analysis depending on the phase. However, discussion of the statistical analysis methods that should be applied to observational datasets within each management phase is limited. To the best of our knowledge, there is a lack of methods to obtain all necessary information simultaneously.

Herein, we first present an overview of several suitable statistical analysis methods for each data analysis objective in the early and late phases of chemical management. Next, using an available river survey dataset, we demonstrate examples of linear regression analyses and discuss the problems associated with not determining the covariate selection procedures based on the data analysis objectives.

## STATISTICAL ANALYSIS METHODS FOR OBSERVATIONAL DATASETS

### Data analyses in the early phase of chemical management

An aim of the early chemical management phase is to identify sites where biological integrity is severely compromised. An effective approach is to develop regression models with different combinations of independent variables and perform model selections for them using Akaike information criterion (AIC; Akaike, 1973) values. The AIC is calculated using the maximum log‐likelihood and the number of estimated parameters of the model; model selection using AIC reveals the regression model, with a high power to predict the dependent variable (i.e., an indicator representing river ecosystem conditions). Models with a relatively low AIC value yield a relatively high predictive power; a larger difference in AIC values among models indicates that the model with a relatively low AIC value has a relatively high predictive power. In addition, statistical machine learning methods, such as support vector regression (Were et al., 2015), have aided in regression model development, especially in cases with nonlinear relationships between the dependent and independent variables, and complex interactions among independent variables. A regression model with a high predictive power developed based on these methods can also be used to derive a risk (hazard) map (Lee & Jetz, 2011). The risk map can help visualize biological conditions over a relatively large area, although careful interpretation is needed when extrapolating model results.

The partial regression coefficients of the models, including a model developed based on AIC model selection, correspond to the strengths of the associations between each environmental factor and the dependent variable. Therefore, the environmental characteristics of sites where biological integrity is compromised can be identified from regression models, thereby allowing the screening of chemicals to be addressed in the late chemical management phase. However, in a model developed based on the predictive power of the dependent variable value, the partial regression coefficients for each independent variable do not reflect the intervention effect sizes of the independent variable values. This is because the small AIC value of a model does not provide a theoretical implication that there is no confounding bias in this model. In other words, the management measures required to restore the river ecosystem to the targeted condition cannot be based on the partial regression coefficient of a model developed according to AIC model selection (i.e., it should not be interpreted as a consistent estimator of its intervention effect size). This is not a weakness of AIC model selection, but rather an inappropriate use of it. A model with the minimum AIC value only has the highest power to predict a dependent variable value in a set of independent variable values, among the candidate models. In addition, although it is not limited to AIC model selection, model complexity selected based on the AIC value changes with the sample size (Shao, 1997). Therefore, it is preferable to avoid relying solely on a statistical model developed based on its predictive power in chemical screening. Screening should be conducted comprehensively, considering other information such as toxicity values derived from laboratory tests and exposure concentrations measured in the field.

### Data analyses in the late phase of chemical management

After selecting the candidate sites where biological integrity is severely compromised and/or screening for chemicals that may need management measures in those sites, the next step is to evaluate the recovery potential of the intervention in the concentration of the targeted chemical(s). This allows a specific discussion of measures to control the environmental concentrations of the targeted chemicals. The term “next step” is used here only for convenience and, in practice, chemical management may sometimes start at this phase.

To estimate intervention effect sizes of concentrations of the targeted chemicals, statistical causal inference methods are required, which differ from statistical analysis methods aimed at predicting the dependent variable value. Statistical causal inference has been applied in ecotoxicological studies; for example, the method of propensity score matching (Austin, 2011) has been used to estimate the effect sizes of insecticides (Yuan et al., 2009). The propensity score is defined as the probability that an individual data point is assigned to a particular treatment (e.g., exposure or nonexposure to insecticides) and estimated using a statistical model (e.g., logistic regression model) with covariates. Thereafter, matching of individuals based on the estimated propensity score is performed to control the confounding bias derived from the nonrandomized assignment of treatment and control groups. If the data analysis objective is to determine the changes in biological conditions caused by the introduction of a management measure, (i.e., if the focal independent variable value can be treated as an ordinal variable such as a binary variable of sites with or without a measure), this statistical method is then appropriate. In addition, structural equation modeling (Grace et al., 2010) has been used to estimate the effect sizes of multiple physical and chemical stressors (Schmidt et al., 2019). This method allows the estimation of the relationships among multiple components, such as ecological networks, and can simultaneously estimate and compare the effect sizes of multiple environmental factors on biological communities. However, there remain cases where an observational dataset with various environmental variables that can be used in this statistical method is available partially because of limited funds for chemical management. Furthermore, a regression model with a set of covariates selected based on the backdoor criterion (Pearl, 1993) has been used to obtain a consistent estimator of the intervention effect size of a trace metal (Takeshita et al., 2020a; see the subsequent section for details of this method). These statistical analysis methods must be used based on the interests of analysts and available dataset properties.

Although in this study, we focused mainly on statistical methods used to analyze nonsystematic observational datasets, previous studies have estimated the intervention effects of trace metal concentrations through well‐designed field experiments (e.g., the so‐called Before–After Control–Impact study; Kotalik et al., 2021). It is also possible to conduct other well‐designed field studies, such as those based on randomized controlled trials (Fisher, 1947).

## CASE STUDY

There are several options for selecting appropriate statistical analysis methods in both early and late phases of chemical management. In this selection process, regression analysis, the most frequently used statistical method in ecotoxicology, was adopted for all three data analysis objectives, namely identifying severely compromised sites, screening chemicals with a high priority for risk assessment, and estimating the intervention effect sizes. Furthermore, we considered that regression analysis is suitable to illustrate the importance of choosing appropriate statistical analysis methods by comparing the data analysis approaches in the early and late phases of chemical management from the perspective of covariate selection. Moreover, for practical reasons, the analyzed dataset did not include enough environmental variables to allow the application of structural equation modeling; we did not identify any plausible threshold to convert chemical concentrations into ordinal variables for applying propensity score methods.

### Dataset

To demonstrate an example of appropriate statistical analysis procedures in the early and late phases of chemical management, we analyzed an observational dataset obtained from the Ministry of the Environment of Japan (2017) with linear regression analysis methods. This dataset included field survey results on river water quality, physical river characteristics, as well as a list of benthic macroinvertebrate species names and their abundance at 45 sites in 14 basins across 8 prefectures of Japan. The survey was conducted from November 2016 to January 2017 by the Ministry of the Environment of Japan owing to concerns regarding the ecological risk of nickel (Ni) in river environments (Ministry of the Environment of Japan, 2017). The free‐ion concentrations of five trace metals (Ni, copper [Cu], zinc [Zn], cadmium [Cd], and lead [Pb]) in the 45 sites predicted using WHAM software (Tipping, 1994) have been previously reported by Takeshita et al. (2019).

We calculated Simpson's diversity index for Insecta in each survey site and set it as the dependent variable in the subsequent statistical analyses. Using this metric, we could detect a decrease in river ecosystem biodiversity caused by, among others, the dominance of taxa insensitive to chemical exposure.

In the present study, we did not focus further on the utility of the diversity index as a biological indicator because it was beyond the scope of this study. However, indicator selection in accordance with the endpoint set in each management phase is a critical issue in field survey design (Mebane et al., 2015; Takeshita et al., 2020a). In addition, in this study, we did not focus on detailed changes in community structure using multivariate analysis methods (e.g., redundancy analysis; Takeshita et al., 2020b). An advantage of using the multivariate analysis methods in ecotoxicological studies is that a high diversity index value does not always reflect the ecosystem integrity; for example, an increase in invasive alien species is undesirable regardless of how the diversity index value increases. However, disadvantages include not having the log‐likelihood required to calculate AIC (Oksanen et al., 2019) and the inability to accurately distinguish between the effects of chemicals and those of other environmental factors, which are correlated with the chemical concentrations, in some of these methods.

We performed all statistical analyses using R 4.0.3 (R Core Team, 2020) and used the R package “MuMIn” ver. 1.43.17 (Bartoń, 2020) for AIC model selection. We also used “emmeans” ver. 1.3.2 (Lenth, 2019) to calculate estimated marginal means (Searle et al., 1980), which are the predicted mean values of the dependent variable when independent variable values are fixed at a specific value (means of each independent variable were used in this study).

### Early phase

#### Methods

We constructed a generalized linear model to identify sites where biological conditions were compromised, as well as the environmental characteristics of the sites. As diversity index values range from 0 to 1, we used Gaussian distribution and logit link function as the probability distribution and link function of the model, respectively.

For the dependent variable set of the regression model, the late phase of chemical management requires careful consideration of environmental factors that should be statistically controlled to estimate the targeted intervention effects. In contrast, this is not as important when developing a regression model with a high predictive power for the dependent variable value. Therefore, our initial full model was a regression model that included most environmental factors available in the Ministry of the Environment of Japan (2017) and Takeshita et al. (2019): nine river water quality parameters (e.g., free‐ion concentrations of five trace metals, pH, and water hardness), four physical river characteristics (e.g., river width and depth, and riverbed sediment type), and two other variables (surveyed month and basin). Subsequently, we excluded Cd and Pb concentrations whose observed maximum concentrations were lower (i.e., statistically negligible) than their chronic toxicity values (e.g., Mebane et al., 2008, 2020) from the initial model. The maximum dissolved and free‐ion concentrations were 0.062 and 0.040 μg/L for Cd, and 0.26 and 0.013 μg/L for Pb, respectively. We also excluded variables in which the software would not return coefficient estimates when they were included as independent variables (e.g., river width and depth) in the model (i.e., variables that did not fit the constructed model). Consequently, the independent variable set of the final full model was: log10‐transformed free‐ion concentrations of trace metals (Ni, Cu, and Zn), total organic carbon (TOC) concentration, pH, water temperature, flow velocity, riverbed sediment, and basin. The TOC concentration was used as an indicator of organic pollution related to domestic wastewater. The analyzed dataset included biological oxygen demand value, and it would be a more suitable organic pollution indicator than TOC. However, biological oxygen demand values reportedly reveal larger intra‐day variation than TOC concentrations (Tanaka et al., 2009). Such an unstable variable should lower the precision of parameter estimation (Spearman, 1904). Moreover, because the 45 survey sites were in the transition zones between semi‐urban and urban areas and not near forest areas, we considered the amount of organic carbon derived from decaying plant material negligible. Therefore, we used TOC concentration as an indicator of organic pollution related to domestic wastewater. Riverbed sediment and basin were categorical variables, and the former had three levels (sand, gravel, and boulder) depending on the representative sediment particle size.

After developing the model, we calculated the AIC values for each of the 512 candidate models, which had different independent variable combinations, and then compared them. We selected the model with the minimum AIC value as the best model. We did not perform model selection based on the statistical significance of each partial regression coefficient, because this approach is theoretically irrelevant to improving the model's predictive power.

#### Results

The model with the minimum AIC value included TOC concentration, free Cu ion concentration, water temperature, riverbed sediment, flow velocity, and basin (Table 1). There was a negative association between TOC concentration and diversity index (Table 2). The free Cu ion concentration was positively associated with the diversity index (Table 2); this result might be influenced by, for instance, outlier free Cu ion concentration data. The free Ni ion concentration was not included in this model (Table 1). It should be noted that this result did not indicate that the intervention effect of Ni concentration was negligible; rather, it indicated that the variable of Ni concentration did not substantially contribute to the predictive power of the model.

**Table 1:** Independent variable set, Akaike information criterion (AIC), and Akaike weights of the top 10 models with the minimum ΔAIC values

Model rank	TOC	Nickel	Zinc	Copper	pH	Water temperature	Riverbed	Flow velocity	Basin	AIC	ΔAIC^a^	Akaike weights
1	+^b^			+		+	+	+	+	−70.68	0.00	0.16
2	+		+	+		+	+	+	+	−68.99	1.69	0.07
3	+			+	+	+	+	+	+	−68.87	1.81	0.07
4	+	+		+		+	+	+	+	−68.72	1.96	0.06
5	+			+			+	+	+	−68.64	2.04	0.06
6	+			+			+		+	−68.01	2.67	0.04
7	+	+		+			+		+	−67.90	2.78	0.04
8	+	+		+			+	+	+	−67.89	2.79	0.04
9	+			+		+	+		+	−67.78	2.90	0.04
10	+		+	+	+	+	+	+	+	−67.16	3.52	0.03

Abbreviation: TOC, total organic carbon.

^a^ ΔAIC indicates differences in AIC values relative to the minimum value.

^b^ “+” indicates factors included in the model.

**Table 2:** Estimated intercept and partial regression coefficients for the independent variables of the model with the minimum Akaike information criterion value

Intercept	TOC	Copper	Water temperature	Riverbed sediment^a^		Flow velocity	Basin^b^		
				Gravel	Boulder		Osawa	Koromo	Suikawa
5.91	−0.54	0.54	−0.15	−0.73	−1.12	1.46	−0.49	−1.20	0.00
(1.23)	(0.15)	(0.19)	(0.09)	(0.44)	(0.40)	(0.85)	(0.79)	(0.70)	(0.55)
*p* < 0.01	*p* < 0.01	*p* = 0.01	*p* = 0.12	*p* = 0.11	*p* = 0.01	*p* = 0.10	*p* = 0.54	*p* = 0.10	*p* = 1.00

Basin^b^									
Kasukawa	Kyuahaku	Ino	Yoshida	Shibue	Kurokawa	Noda	Tsuho	Takizawa	Nigori
−0.55	1.00	−1.47	−0.45	−0.77	0.04	0.55	−0.13	−3.45	−2.81
(0.68)	(0.54)	(0.78)	(0.80)	(0.67)	(0.61)	(0.91)	(0.92)	(0.94)	(0.96)
*p* = 0.43	*p* = 0.08	*p* = 0.07	*p* = 0.58	*p* = 0.27	*p* = 0.94	*p* = 0.94	*p* = 0.88	*p* < 0.01	*p* < 0.01

*Note*: Values in parentheses are standard errors of the estimates.

Abbreviation: TOC, total organic carbon.

^a^ Coefficients for “Gravel” and “Boulder” represent the difference from the reference sediment category (sand).

^b^ Coefficients for each basin represent the difference from the reference basin (Yata River).

#### Implication for the next chemical management phase

**Figure 1:**
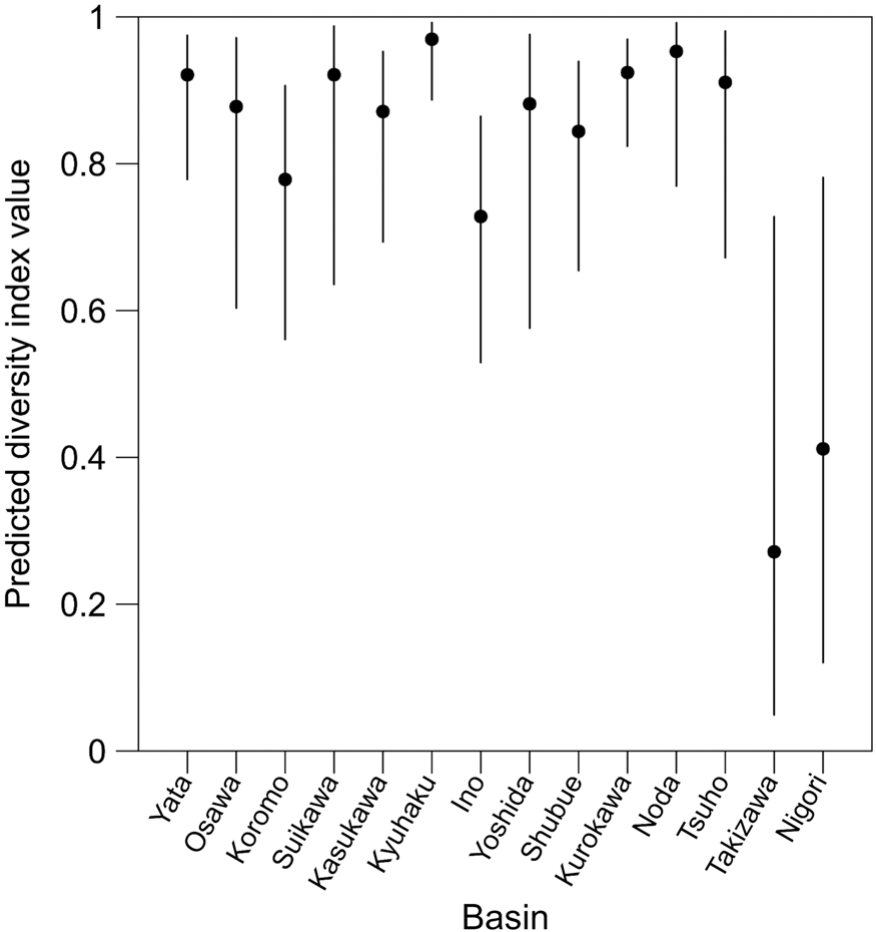
Estimated marginal means (black dots) and their 95% confidence intervals (bars) for Simpson's diversity index for Insecta in 14 basins in Japan. We used the parameter estimates of the model with the minimum AIC value and the R package “emmeans” to depict this figure

**Figure 2:**
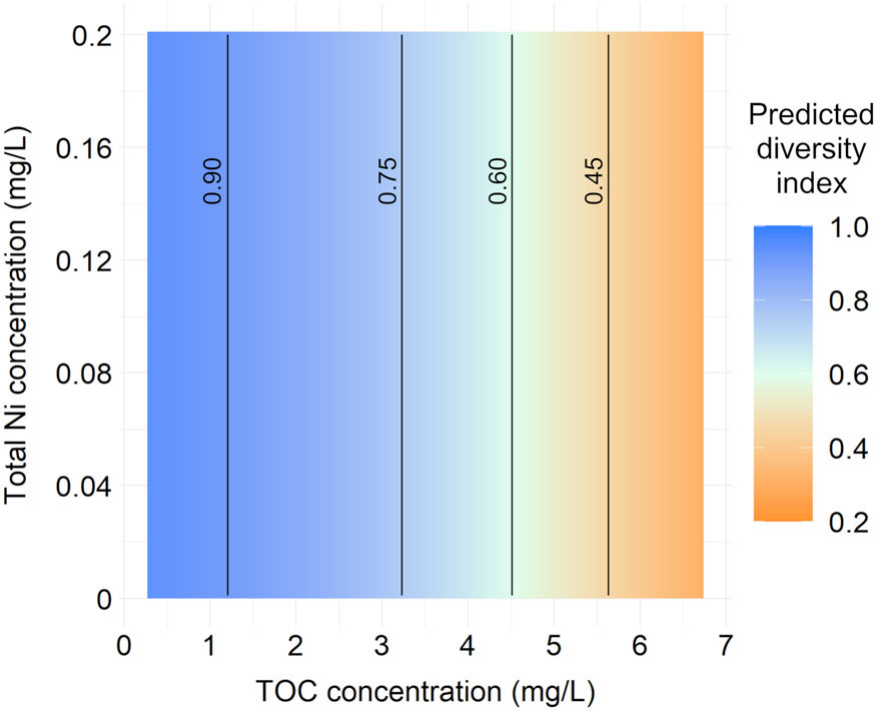
Heatmap and contour lines of the predicted Simpson's diversity index for Insecta, at TOC concentrations of 0.3–6.7 mg/L and total Ni concentrations of 0.001–0.2 mg/L. The prediction was based on the estimated marginal means calculated using the parameter estimates of the model with the minimum AIC value. In chemical management, it is often more useful to present the outputs of data analysis based on their total concentrations rather than based on free‐ion concentrations or dissolved concentrations. Therefore, we predicted the Simpson's diversity index by relating it to the concentrations of TOC and total Ni through metal speciation calculation with WHAM and numerical calculation with R. Details of this procedure are provided in the Supporting Information

A key goal of the statistical analysis in the early chemical management phase is to determine where serious damage to ecosystems has occurred using parameter estimates of the model selected based on the AIC values. The diversity index values were particularly small (but there were relatively large within‐basin variations) in the two basins in Yamanashi Prefecture (Takizawa and Nigori basins; Figure 1). Sites with higher TOC concentrations also had lower predicted diversity index values (Figure 2). Consequently, sites with high TOC concentrations or those in Yamanashi Prefecture basins were determined to be candidate sites to implement management measures for the conservation of ecosystems with high priority. However, Takizawa and Nigori basins had considerably larger negative partial regression coefficients than other basins (Table 2), suggesting that the biological conditions in these two basins were compromised by unmeasured factors. Therefore, it is necessary to further examine if aquatic insects in these two basins can be recovered by implementing management measures. For example, these basins may initially be unfavorable habitats for aquatic insects because of macroscale environmental factors such as climate.

Another objective in the early phase, that is, screening for chemicals to be investigated in detail and their prioritization, can be conducted based on the results of AIC model selection. The results suggest that it is necessary to estimate the intervention effect size of TOC concentrations while considering future implementation of management measures for organic pollution.

In addition, the use of only the AIC model selection results for chemical screening should be avoided; a comparison of observed chemical concentrations with their toxicity values and environmental quality benchmarks are also essential in chemical screening. This is because the partial regression coefficients for each independent variable in the model selected based on AIC values cannot be basically interpreted as consistent estimators of their intervention effect sizes. For example, the observed positive association between Cu concentration and diversity index does not indicate that the intervention effect of Cu concentration was positive, that is, a reduction in Cu concentration results in a decrease in the diversity of aquatic insect communities. This is contrary to the findings of a previous study (e.g., Leland et al., 1989). In addition, in the analyzed dataset, the Cu concentration was low (the median value was 0.0048 μg/L), and therefore, the negative effects on diversity were undetectable, although the maximum free Cu ion concentration (0.16 μg/L) might have been sufficient to affect a few insect species sensitive to Cu exposure (Mebane et al., 2020). Consequently, we did not estimate the intervention effect size of free Cu ion concentration in the next subsection.

Next, we considered whether there is a need to estimate the intervention effect size of Ni concentrations. One reason is that the field surveys by the Ministry of the Environment of Japan were conducted because of Ni contamination concerns in riverine environments. In the analyzed dataset, the maximum and median concentrations of free Ni ion were 183.6 and 5.0 μg/L, respectively. The maximum concentration was comparable to the chronic toxicity value (Mebane et al., 2020; Nakanishi & Tsunemi, 2008). Moreover, although the benchmark for Ni has not yet been established in Japan, the Priority Substances Daughter Directive (2013/39/EU) under the Water Framework Directive set the annual average environmental quality standard for Ni and its compounds at 4 μg/L (in bioavailable forms) for inland surface waters. In addition, the strength of correlations between other independent variables also influences whether an independent variable is included in the minimum AIC model, and trace metal concentrations are generally positively correlated with each other. Therefore, considering the original aim of the field surveys conducted by the Ministry of the Environment of Japan, we decided to estimate the intervention effect size of free Ni ion concentrations although Ni concentration was not included in the minimum AIC model.

### Late phase

#### Methods

In our late phase statistical analysis example, we estimated the intervention effect of TOC and Ni concentrations based on the results of the early phase data analysis and the comparison of observed chemical concentrations with their toxicity values and environmental quality benchmarks. To estimate the intervention effect sizes of TOC and Ni concentrations, we adopted a regression analysis approach and determined a set of independent variables of the regression model based on the backdoor criterion. This criterion enabled us to determine a set of independent variables required to obtain a consistent estimator of the targeted intervention effects from the partial regression coefficients while removing some estimation bias, such as that derived from confounders (Pearl et al., 2016). For the analyzed dataset, the set of independent variables that satisfied the backdoor criterion to estimate the intervention effects of TOC and Ni concentrations were log_10_‐transformed concentrations of free Ni, Cu, and Zn ion and TOC concentrations; pH; flow velocity; riverbed sediment; and basin (Supporting Information). Model selection should not be performed after determining the independent variable set based on the backdoor criterion. We noted that water temperature was an unnecessary variable when estimating the targeted intervention effects; this variable was included in the model with the minimum AIC value (Table 1). We used Gaussian distribution and logit link function as the probability distribution and link function of the model, respectively.

#### Results

**Figure 3:**
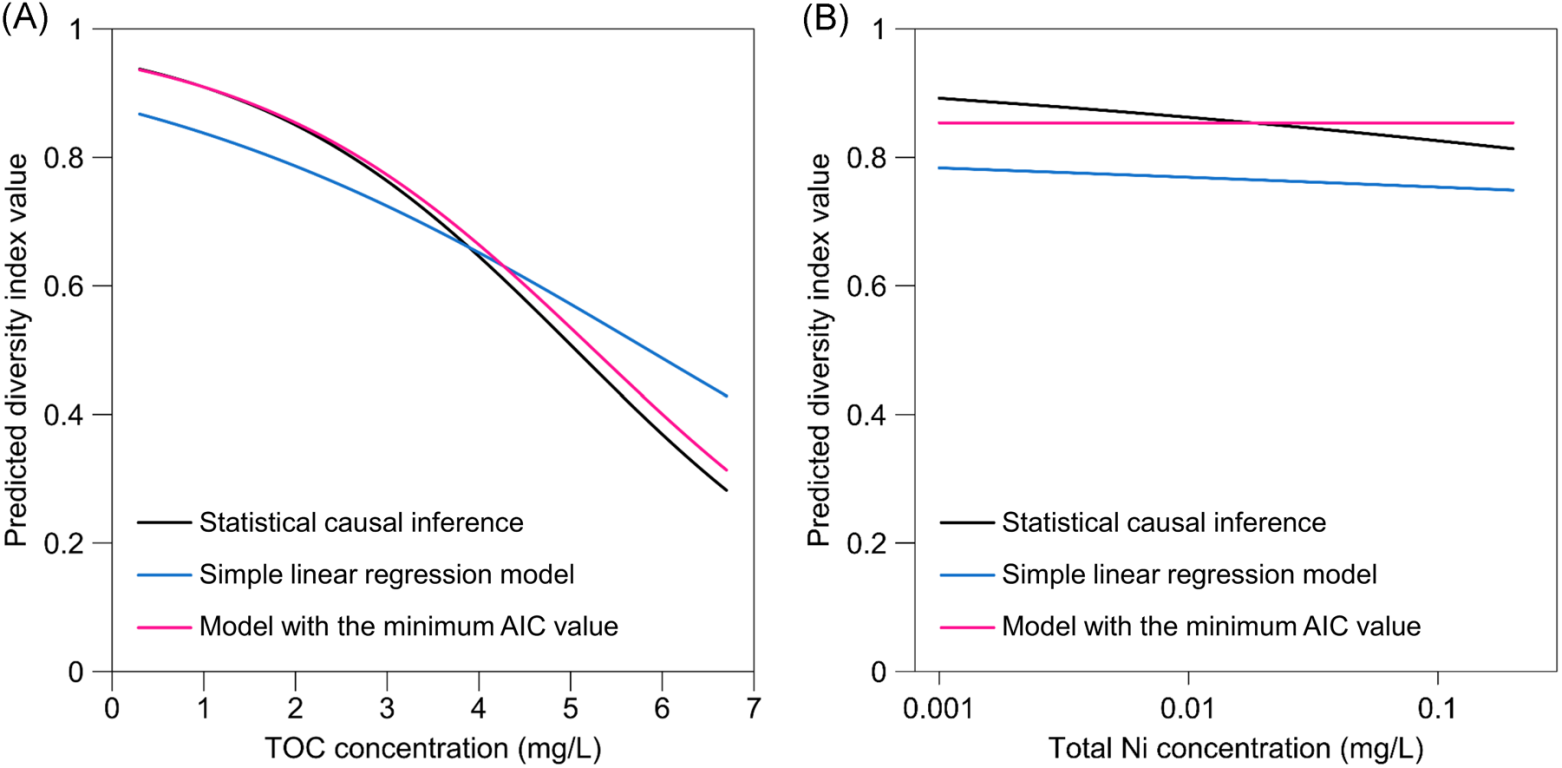
Predicted Simpson's diversity index for Insecta at (A) TOC concentrations of 0.3–6.7 mg/L and (B) total Ni concentrations of 0.001–0.2 mg/L. The prediction was based on the estimated marginal means calculated using the parameter estimates of the following three regression models: multiple linear regression model with the independent variable set satisfying the backdoor criterion (i.e., statistical causal inference, solid black line), simple linear regression model (solid blue line), and the model with the minimum AIC value (solid pink line). Details of the procedure to depict this figure are provided in the Supporting information

In the regression model described here, the partial regression coefficients for TOC and Ni concentrations (Table 3) corresponded to consistent estimators of their intervention effect sizes on the diversity index. The reduction in TOC concentration had a substantial effect on diversity index recovery (Figure 3A; Table 3). The reduction in free Ni ion concentration slightly affected diversity index recovery (Figure 3B); however, its *p*‐value was 0.29 (Table 3). The statistical significance (*p*‐value) of partial regression coefficients depends largely on sample size. Moreover, for the analyzed dataset, multicollinearity among the independent variables would reduce the precision of the partial regression coefficient for free Ni ion concentration and consequently influence its *p*‐value (Table S1). The partial regression coefficients correspond to consistent estimators of the targeted intervention effect sizes by satisfying the backdoor criterion, which is a different issue from the decrease in the precision of estimated partial regression coefficients owing to multicollinearity. Therefore, considering the small sample size of the analyzed dataset, ignoring the intervention effect of Ni concentration based on the moderate *p*‐value is not recommended, and additional surveys would require management measures for Ni concentration to be decided.

**Table 3:** Estimated intercept and partial regression coefficients for the independent variables and their *p*‐values, of the models with a set of independent variables satisfying the backdoor criterion to estimate the intervention effect in total organic carbon and nickel concentrations on Simpson's diversity index

Intercept	TOC	Nickel	Zinc	Copper	pH	Riverbed sediment^a^		Flow velocity	Basin^b^		
						Gravel	Boulder		Osawa	Koromo	Suikawa
2.97	−0.57	−0.28	0.23	0.55	0.22	−0.71	−1.02	1.10	−0.90	−1.01	0.09
(6.46)	(0.20)	(0.26)	(0.34)	(0.25)	(0.81)	(0.47)	(0.42)	(0.92)	(0.92)	(0.83)	(0.61)
*p* = 0.65	*p* < 0.01	*p* = 0.29	*p* = 0.49	*p* = 0.04	*p* = 0.79	*p* = 0.14	*p* = 0.02	*p* = 0.24	*p* = 0.34	*p* = 0.24	*p* = 0.88

Basin^b^									
Kasukawa	Kyuahaku	Ino	Yoshida	Shibue	Kurokawa	Noda	Tsuho	Takizawa	Nigori
−1.08	0.73	−1.58	−1.05	−1.06	0.13	0.57	−0.07	−2.30	−2.20
(0.78)	(0.53)	(0.91)	(0.76)	(0.76)	(0.68)	(1.09)	(1.02)	(0.89)	(0.94)
*p* = 0.18	*p* = 0.18	*p* = 0.09	*p* = 0.18	*p* = 0.18	*p* = 0.85	*p* = 0.61	*p* = 0.95	*p* = 0.02	*p* = 0.03

*Note*: Values in parentheses are standard errors of the estimates.

Abbreviation: TOC, total organic carbon.

^a^ Coefficients for “Gravel” and “Boulder” represent the difference from the reference sediment category (sand).

^b^ Coefficients for each basin represent the difference from the reference basin (Yata River).

#### Comparison with the results from inappropriate covariate selection procedures

We compared the estimated intervention effect sizes of TOC (major driver) and free Ni ion (minor driver) concentrations with their “strengths of association” derived from two other regression models: a simple linear regression model and multiple linear regression models developed based on AIC model selection. A simple linear regression model was used for comparison as the most extreme case where no statistical control for confounders was made.

For the TOC concentration case, the strength of association derived from the simple linear regression model and the model with the minimum AIC differed from the estimated intervention effect size of the statistical causal inference (Figure 3A). However, the differences seemed to be small compared with the Ni concentration case (Figure 3B). This suggests that, in our case study, even if the effect sizes of other limiting factors of insect diversity were spuriously included in the effect of TOC concentrations caused by uncontrolled confounders, the deviation from the actual effect size of TOC was small because the effect size of TOC concentration was relatively large compared with these other factors. We emphasize that this result does not indicate that AIC model selection is a theoretically appropriate analytical procedure to estimate the intervention effect size of a major driver. For the Ni concentration case, the difference between the model developed based on AIC model selection and the statistical causal inference is reasonable (Figure 3B), at least to the extent that it could affect the Ni management plan (AIC model selection; negligible substance vs. statistical causal inference; substance with a slight but steady negative effect).

## CONCLUSIONS

In this article, we presented an overview of important data analysis objectives in the early and late phases of chemical management, as well as some appropriate statistical analysis methods for observational datasets associated with each objective. We also showed examples of linear regression analysis procedures using an available dataset derived from field surveys of Japanese rivers. The issues that occurred in our case study, such as the exclusion of minor drivers during AIC model selection (i.e., the possibility of overlooking Ni during chemical screening), do not always occur in other data analyses. However, our results demonstrate the dangers of relying on one regression model for achieving all of the analytical objectives: identifying severely compromised sites, screening chemicals with a high priority for risk assessment, and estimating the intervention effect sizes. To identify sites where biological conditions are compromised in the early phase of chemical management, we should develop a regression model that focuses on the model's predictive power for the dependent variable (biological indicator) value. Meanwhile, to discuss the content of specific management measures in the late phase, including examining the validity of the extant environmental quality standard (Crane et al., 2007; Matsuzaki, 2011; Peters et al., 2014), estimates of intervention effect sizes from statistical causal inference methods are required. Appropriate management measures cannot be determined if an inappropriate covariate selection method of regression analysis is chosen without considering what has to be estimated from observational datasets. In ecotoxicology, some studies have performed AIC model selection although the data analysis objective was not to predict the dependent variable value but to examine the presence of the association between the dependent and independent variables (e.g., Takeshita et al., 2019). Furthermore, Budge et al. (2015) performed model selection based on the statistical significance of each partial regression coefficient although estimating the intervention effect size was the goal of the study. Such a model selection approach based on statistical significance is theoretically irrelevant for both estimating the intervention effect size and improving the model's predictive power. In other words, as the null hypothesis in this statistical hypothesis test is that the partial regression coefficient is zero, its *p*‐value does not determine whether the explanatory variable is a confounder or not, or whether it is useful in predicting the value of the target variable. Our study will facilitate future research on chemical management in choosing the appropriate covariate selection method of regression analysis for observational datasets.

## CONFLICT OF INTEREST

The authors declare that there are no conflicts of interest.

## Supporting information

Details of the independent variable selection procedure and procedures for depicting figures are provided.

## Data Availability

All datasets supporting this manuscript were obtained from previous studies (Ministry of the Environment of Japan, 2017; Takeshita et al., 2019).
