# Supplementary material for: What do we want to estimate from observational datasets? Choosing appropriate statistical analysis methods based on the chemical management phase
Source: Integr Environ Assess Manag. 2022 Jan 12;18(5):1414–22. doi: 10.1002/ieam.4564 (PMC9539851; doi:10.1002/ieam.4564)
Supplement: Supplementary file 1 — Details of the independent variable selection procedure and procedures for depicting figures are provided. [file IEAM-18-1414-s001.docx]

**Supplementary Material for “What do we want to estimate from observational datasets? Choosing appropriate statistical analysis methods based on the chemical management phase”**

**INDEPENDENT VARIABLE SELECTION BASED ON THE BACKDOOR CRITERION AND THE WORKING CAUSAL DIAGRAM**

Here we explain the independent variable selection procedure based on the backdoor criterion for estimating the intervention effect size of free nickel (Ni) ion and total organic carbon (TOC) concentrations. Takeshita et al. (2020) provides a detailed explanation for the backdoor criterion and the background of spurious associations derived from a confounder using a pseudo-dataset. The authors have also explained the independent variable selection procedure for estimating the intervention effect size of free Ni ion concentrations with the dataset of the Ministry of the Environment of Japan (2017); however, they did not examine the independent variable selection procedure for estimating the intervention effect size of TOC concentrations in-depth. Therefore, we discuss the independent variable selection procedure and address such information gaps.

***An overview of the backdoor criterion***

In linear regression analyses, when an independent variable set *S* satisfies the following two conditions (the backdoor criterion; Pearl, 1993; Hernán & Robins, 2020), the partial regression coefficient for the target-independent variable (treatment variable) corresponds to a consistent estimator of its intervention size on the dependent variable (outcome variable). The first condition is that all backdoor paths for treatment and outcome variables are blocked by *S*. Here, we used an expression of graphical rules of causal diagrams to describe these two criteria; the term “backdoor path” applies to paths that have input to both treatment and outcome variables. The term “block” is also based on graphical rules of causal diagrams; the term “conditioning” may be more familiar from an expression in the field of statistics. The second condition is that none of the paths leading from the treatment variable are blocked by *S*. In other words, any independent variable included in *S* should not be a descendant variable of the treatment variable (the so-called intermediate variables; Rosenbaum, 1984).

***The backdoor criterion for estimating the intervention effect size of the free Ni ion concentrations***

Based on the working causal diagram (Fig. S1) and the backdoor criterion, we determined the regression model providing the intervention effect size of the free Ni ion concentrations on the aquatic insect diversity. In this study, the backdoor paths between free Ni ion concentrations and aquatic insects can be roughly classified into four types: those related to (1) the variable “basin,” (2) the variable “season,” (3) the variable “urbanization,” and (4) the metal speciation in river water (Fig. S1).

In our working causal diagram, the variable “basin” is expected to function as a surrogate variable describing basin-specific variations in latitude, altitude, climate, and geological features; the within-basin variations in these factors were negligibly small (Ministry of the Environment of Japan, 2017). We included the variable “basin” in the model to block the backdoor paths leading from this variable (e.g., free Ni ion concentrations $\leftarrow$ water temperature $\leftarrow$ basin $\to$ flow velocity $\to$ aquatic insects; Fig. S1). In addition, the backdoor paths leading from the variable “season” (e.g., free Ni ion concentrations $\leftarrow$ dissolved metal concentrations $\leftarrow$ economic activity $\leftarrow$ season $\to$ precipitation $\to$ flow velocity $\to$ aquatic insects; Fig. S1) could be regarded as having already been blocked because each survey site was surveyed only once during the winter season (Ministry of the Environment of Japan, 2017).

For the backdoor paths relating to the variable “urbanization,” we were unable to determine the plausible indicator describing the extent of urbanization (and economic activity). Therefore, we decided to statistically control five descendant variables (flow velocity, riverbed sediment, pH, mineral components, and unmeasured factors being the descendant variable of economic activity) of the variable “urbanization” to block the backdoor paths leading from it. Among these, we did not include the variable “mineral components” in the model because the direct effects of calcium, magnesium, and sodium on aquatic insects could be assumed to be negligibly small considering the observed value ranges in the analyzed dataset. Concerning the unmeasured factors being the descendant variable of economic activity, pesticides and habitat degradation, such as river-improvement work, are known to be critical factors impairing the diversity of stream invertebrates. However, the field surveys by the Ministry of the Environment of Japan were conducted during the winter season to minimize the effects of pesticides on aquatic insects. In Japan, environmental concentrations of pesticides in winter tend to be very low compared with those in other seasons (Hano et al., 2019). Moreover, the survey sites have been selected to minimize differences in habitat conditions (e.g., cross-river structures and riparian vegetation) as much as possible (Ministry of the Environment of Japan, 2017). We thus considered that the unmeasured factors being the descendant variable of economic activity could be negligible by assuming that their effects on aquatic insects are relatively small compared with other factors.

Finally, for the backdoor paths relating to the metal speciation in river water, all of them (e.g., free Ni ion concentrations $\leftarrow$ dissolved metal concentrations $\leftarrow$ pH $\to$ aquatic insects; Fig. S1) were already blocked, including by the variables “basin,” “flow velocity,” “riverbed sediment,” and “pH” in the process of blocking other backdoor paths.

***The backdoor criterion for estimating the intervention effect size of the TOC concentrations***

The backdoor paths between TOC concentrations and aquatic insects can also be classified into four types, the same as those between free Ni ion concentrations and aquatic insects. In other words, the backdoor paths can be blocked when we included the seven variables (free ion concentrations of Ni, Cu, and Zn, pH, flow velocity, riverbed sediment, and basin) in the model under the assumption that the unmeasured factors being the effects of the descendant variable of economic activity were negligibly small compared with other factors.

The first condition of the backdoor criterion for estimating the intervention effect size of the TOC concentrations can be satisfied as noted above; however, the variable “free trace metal ion” is the intermediate variable of “organic pollution.” Including both free trace metal ion and TOC concentrations in the model violates the second condition of the backdoor criterion for estimating the intervention effect size of TOC concentrations. Nevertheless, the metal speciation calculation using the Windermere Humic Aqueous Model (WHAM) software (Tipping, 1994) revealed that the variations in dissolved organic carbon (DOC) concentrations have little effect on the speciation of Ni and zinc (Zn) in the average water quality conditions of the analyzed dataset (Fig. S2A and S2B). These results indicated that in the analyzed dataset, the path leading from the variables “organic pollution” to “free ion concentrations of Ni and Zn” was virtually ignorable. DOC concentrations appeared to have moderate effects on the speciation of Cu compared with Ni and Zn, especially when DOC concentrations were lower than 0.5 or 0.6 mg/L (Fig. S2C). However, the observed DOC concentrations were 0.7 mg/L or more in 39 out of 45 survey sites in the Ministry of the Environment of Japan (2017). This suggested that the path leading from the variables “organic pollution” to “free Cu ion concentrations” can also be virtually ignorable. Then, we consider that a similar relationship is made out not only DOC but also TOC because DOC and TOC concentrations were highly correlated in the analyzed dataset (Pearson’s correlation coefficient *r* = 0.99).

Consequently, we concluded that the partial regression coefficients for Ni and TOC concentrations of a multiple regression model with the above-determined independent variables (free ion concentrations of Ni, Zn, and Cu, TOC concentrations, pH, flow velocity, riverbed sediment, and basin) can be regarded as the consistent estimators for their intervention effect sizes on aquatic insect diversity.

**SUPPLEMENTAL FIGURES**


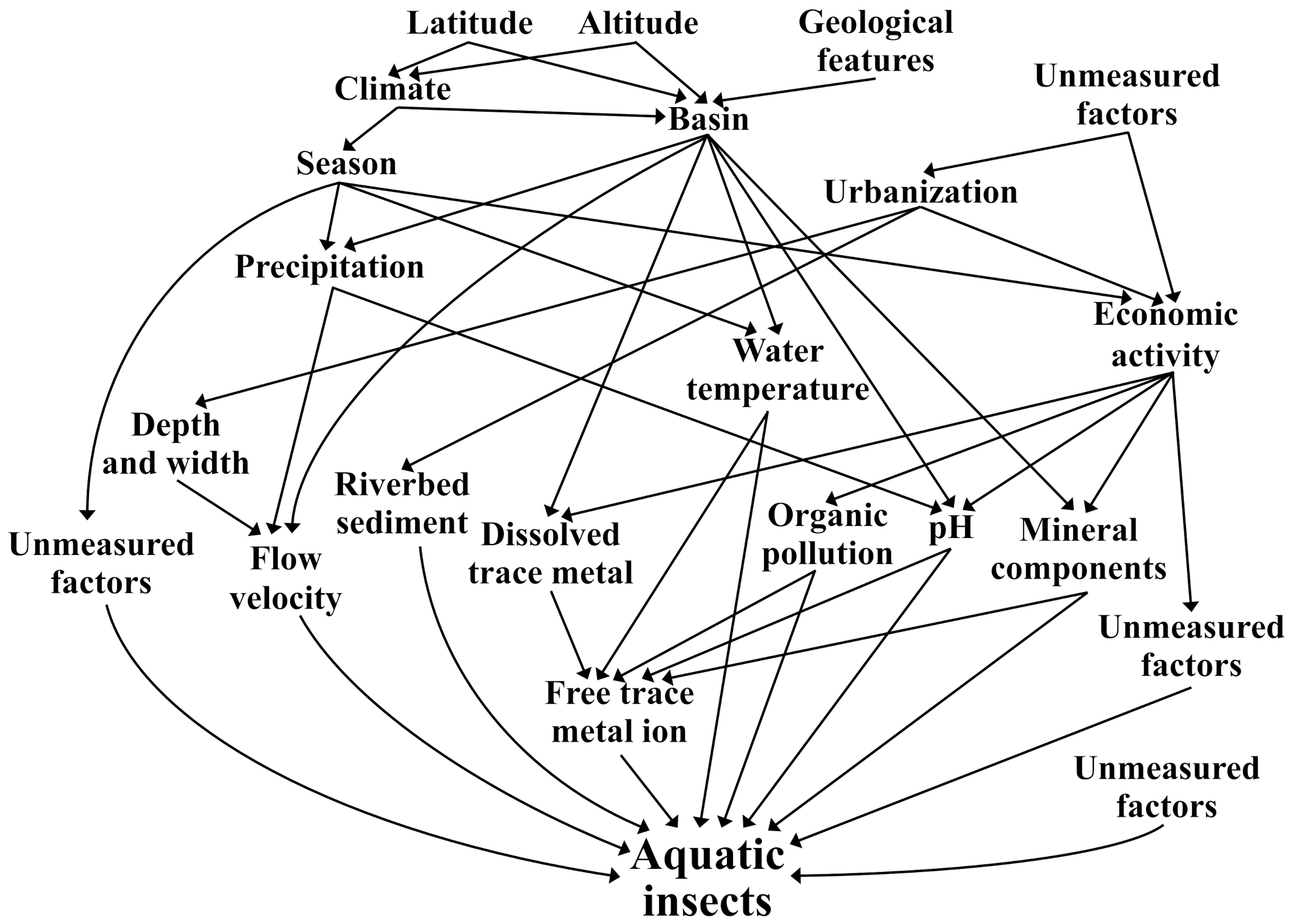


**Figure. S1:** Working causal diagram for aquatic insects and environmental factors. This diagram was developed considering causal structures of the data-generating mechanisms behind the dataset of Ministry of the Environment of Japan (2017) and ecotoxicological, biological, and ecological domain knowledge through literature surveys (see Takeshita et al., 2020 for details). The arrows (i.e., paths) lead from “cause” to “effect.” Causes precede effects. The cause-and-effect relationships depicted in this diagram do not always denote the mathematical input-output relationships originally introduced by Pearl (2009). Factors and paths of a diagram are likely different among studies, our diagram should not be used in other studies without careful examination of the data-generating mechanisms behind the datasets of each study. Our diagram is acyclic; in other words, we did not assume any long-time-scale feedback effects.


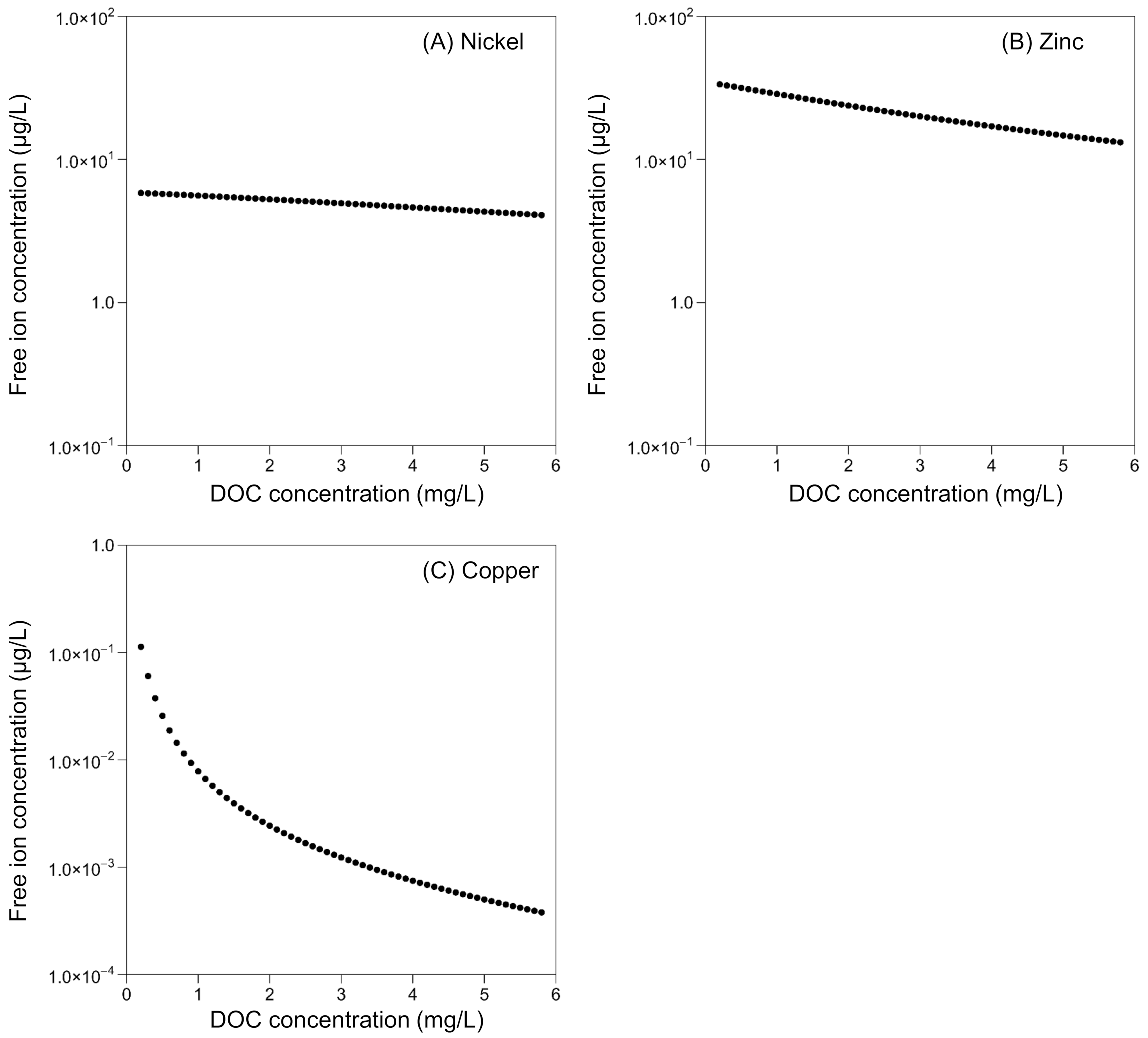


**Figure S2:** Predicted free ion concentrations of (A) nickel, (B) zinc, and (C) copper, for DOC concentrations of 0.2–5.8 mg/L (in 0.1 mg/L increments). This range of DOC concentrations was the observed range in the dataset of the Ministry of the Environment of Japan (2017). The metal speciation calculation was performed using the WHAM software. Input parameters other than DOC concentrations were fixed at the median values of the dataset of the Ministry of the Environment of Japan (2017); particularly, the input values (median) of dissolved concentrations of Ni, Zn, and Cu were 6.957 μg/L, 41.458 μg/L, 1.098 μg/L, respectively. We assumed the DOC concentrations consisted of 100% fulvic acid (Dwane & Tipping, 1998).

**SUPPLEMENTAL TABLE**

**Table S1.** Generalized variance inflation factors adjusted for degrees of freedom for the independent variables of the regression model satisfying the backdoor criterion for estimating the intervention effect sizes of TOC and Ni concentrations on the diversity index. The original calculation was performed by Takeshita et al. (2020) using the R package “car” ver. 3.0.2 (Fox & Weisberg, 2011).

| Independent variable | degrees of freedom | Generalized variance inflation factors |
| --- | --- | --- |
| Free-ion concentrations |  |  |
| Nickel | 1 | 2.10 |
| Zinc | 1 | 2.26 |
| Copper | 1 | 2.00 |
| pH | 1 | 2.41 |
| Total organic carbon | 1 | 2.80 |
| Riverbed sediment | 2 | 1.38 |
| Flow velocity | 1 | 2.32 |
| Basin | 13 | 1.32 |

**PROCEDURE FOR DEPICTING FIGURES** **2 AND 3**

In chemical management, it is often more useful to present the outputs of data analysis based on their total concentrations, rather than based on free-ion concentrations or dissolved concentrations. Therefore, we predicted Simpson’s diversity index by relating it to the concentrations of TOC and total Ni.

We used the parameter estimates from regression models with free metal ion concentrations, the WHAM software, R (R Core Team, 2020), and the R package “emmeans” (Lenth, 2019) to calculate the estimated marginal means (Searle, 1980) for the diversity index values based on the procedures described in the following paragraph. Subsequently, we set the range of TOC concentrations and total Ni concentrations as 0.3–6.7 mg/L and 0.001–0.2 mg/L, respectively. These ranges were determined considering the extent to which the predicted free Ni ion concentrations did not exceed those observed in the field surveys conducted by the Ministry of the Environment of Japan (2017). This avoided any extrapolation of the regression model results.

In Fig. 2, the free Ni ion concentrations for each combination of TOC and total Ni concentrations were predicted using WHAM. For the input amounts of colloidal humic acid and fulvic acid to WHAM, the DOC concentrations at the given TOC concentrations were first predicted using the “emmeans” package, using parameter estimates of the simple linear regression model for TOC and DOC concentrations constructed from the observational dataset (Ministry of the Environment of Japan, 2017). We assumed the predicted DOC concentrations consisted of 100% fulvic acid (Dwane & Tipping, 1998). Other input parameters, such as water temperature, pH, dissolved Ca, Mg, Na, Cd, Cu, Pb, and Zn concentrations, were used as mean values of the analyzed dataset. Finally, diversity index was predicted using the predicted free Ni ion concentrations, original TOC concentration (i.e., values used to predict DOC concentrations), and the parameter estimates of the model with the minimum Akaike information criterion value (Fig. 2). Thus, this prediction included the metal speciation calculation completed with WHAM. For example, reducing the TOC concentration mitigated its negative effect on aquatic insects but also increased the negative effect of free Ni ion concentration through metal speciation.

Related to Figs. 3A and 3B, the prediction of free Ni ion concentrations using WHAM was performed only in Fig. 3B. The input fulvic acid value was 1.7 mg/L, which was the predicted DOC concentration that corresponded to the mean TOC concentration of the analyzed dataset (i.e., 2.0 mg/L). For Fig. 3A, we did not use WHAM, and the free Ni ion concentration was fixed at 17.4 μg/L, the mean value of the analyzed dataset. Estimated marginal means for the diversity index shown in Fig. 3 were then calculated using these values and the parameter estimates of the four models.

**REFERENCES IN THE SUPPLEMENTARY MATERIAL**

Dwane GC, Tipping E. 1998. Testing a humic speciation model by titration of copper-amended natural waters. *Environ Int* 24:609–616.

Fox J, Weisberg S, 2011. An R companion to applied regression (2nd ed). Thousand Oaks (CA): Sage Publications. 472p.

Hano T, Ito K, Ohkubo N, Sakaji H, Watanabe A, Takashima K, Sato T, Sugaya T, Matsuki K, Onduka T, Ito M, Somiya R, Mochida K. 2019. Occurrence of neonicotinoids and fipronil in estuaries and their potential risks to aquatic invertebrates. *Environ Pollut* 252:205–215

Hernán MA, Robins JM. 2020. Causal Inference: what if. Boca Raton (FL): Chapman & Hall/CRC. 302p.

Lenth R. 2019. emmeans: estimated marginal means, aka least-squares means. [2021 March 7]. https://CRAN.R-project.org/package=emmeans

Ministry of the Environment of Japan. 2017. Report on the research for the consideration of the environmental standards for the conservation of aquatic organisms (in Japanese with English abstract). Tokyo (JP): Ministry of the Environment of Japan.

Pearl J. 1993. Comment: graphical models, causality and intervention. *Stat Sci* 8:266–269.

Pearl J. 2009. Causality: Models, Reasoning, and Inference (2nd ed). New York (NY): Cambridge University Press. 484p.

R Core Team. 2020. R: A language and environment for statistical computing. [2021 March 7]. https://www.R-project.org/

Rosenbaum PR. 1984. The consequences of adjustment for a concomitant variable that has been affected by the treatment. *J R Stat Soc Ser A* 147:656–666.

Searle SR, Speed FM, Milliken GA. 1980. Population marginal means in the linear model: an alternative to least squares means. *Am Statistician* 35:216–221.

Takeshita KM, Hayashi TI, Yokomizo H. 2020. The effect of intervention in nickel concentrations on benthic macroinvertebrates: A case study of statistical causal inference in ecotoxicology. *Environ Pollut* 265:115059.

Tipping E. 1994. WHAM—a chemical equilibrium model and computer code for waters, sediments, and soils incorporating a discrete site/electrostatic model of ion-binding by humic substances. *Comput Geosci* 20:973–1023.
